# Supplementary figures and images for: Microbes within the building envelope—a case study on the patterns of colonization and potential sampling bias
Source: PeerJ. 2023 Nov 17;11:e16355. doi: 10.7717/peerj.16355 (PMC10658902; doi:10.7717/peerj.16355)

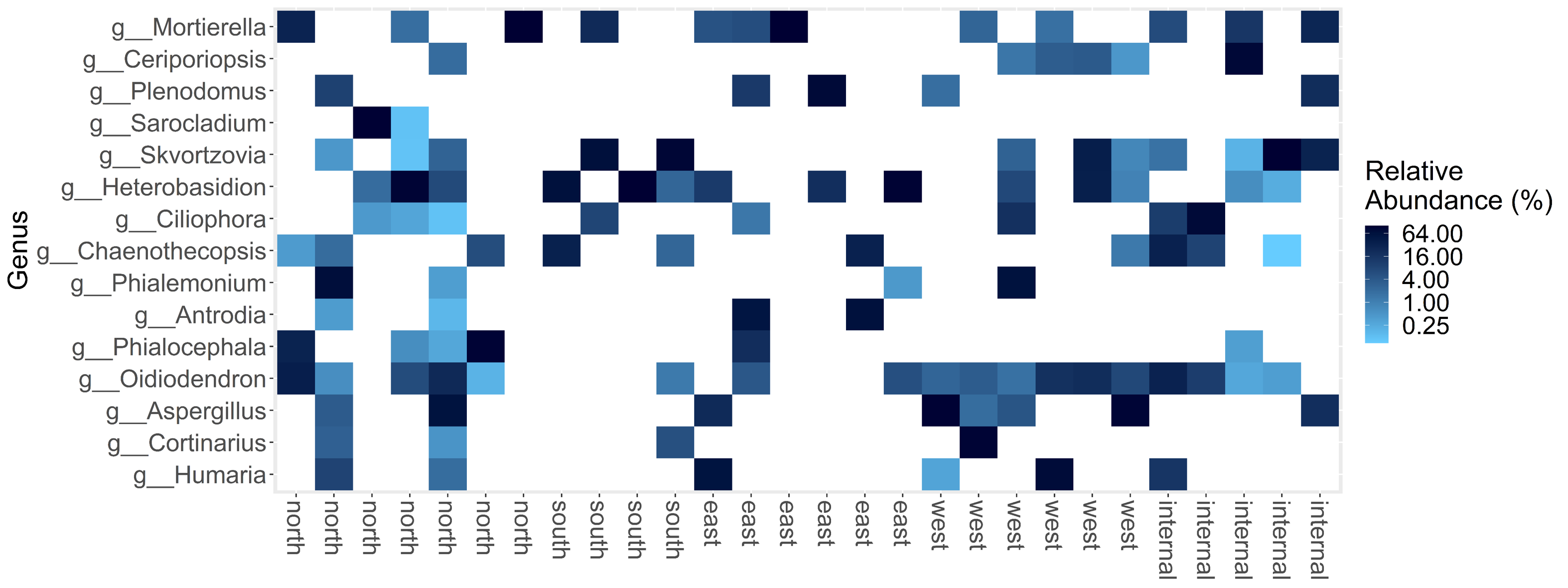

Supplement: Supplemental Information 1 — Darker colour represents higher relative abundance in percentage. [file peerj-11-16355-s001.png]

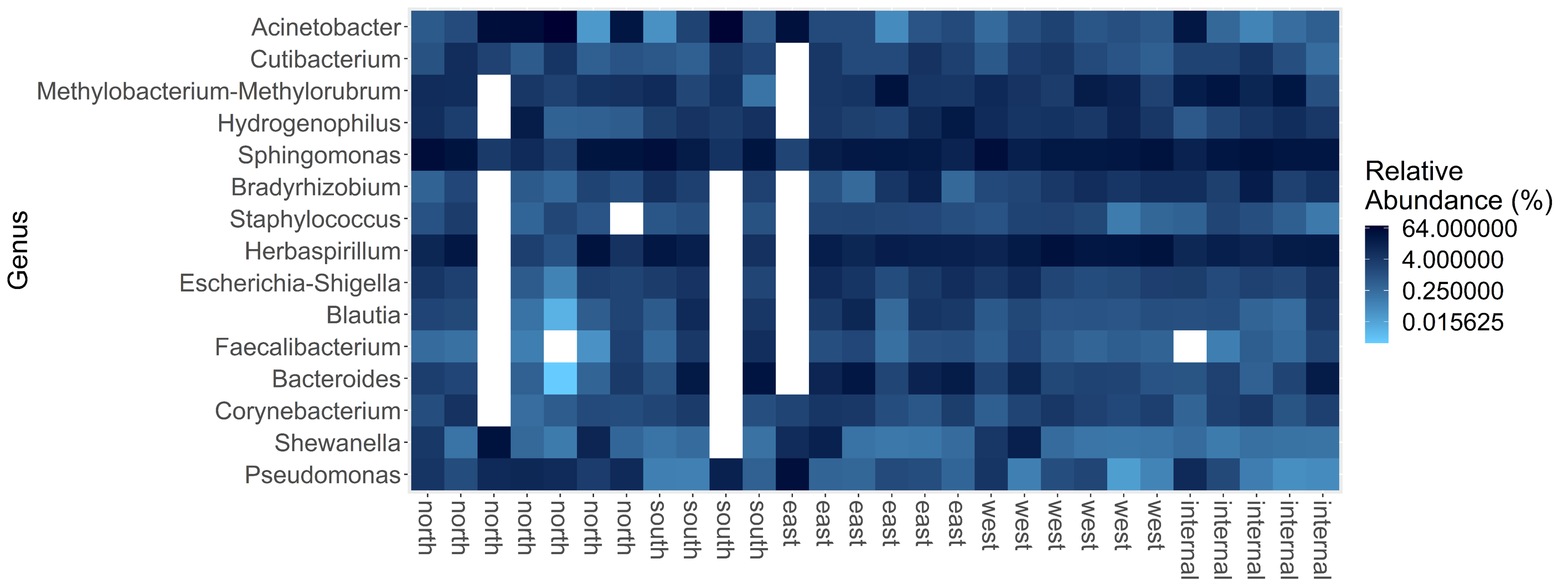

Supplement: Supplemental Information 2 — Darker colour represents higher relative abundance in percentage. [file peerj-11-16355-s002.png]

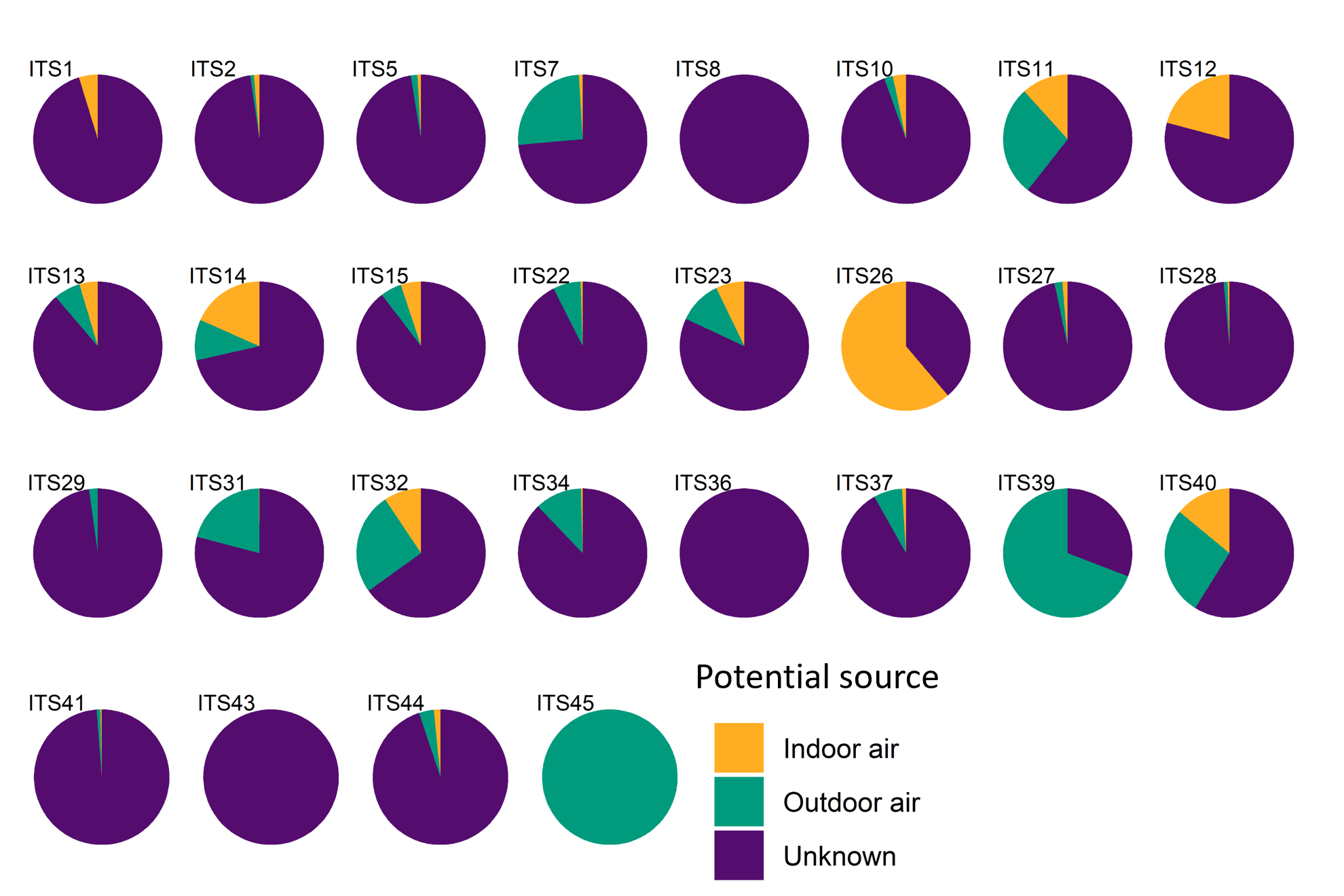

Supplement: Supplemental Information 3 — Sources were labelled as indoor air and outdoor air. Where ASVs didn’t fall in to these categories, they were labeled as coming from an unknown source. [file peerj-11-16355-s003.png]

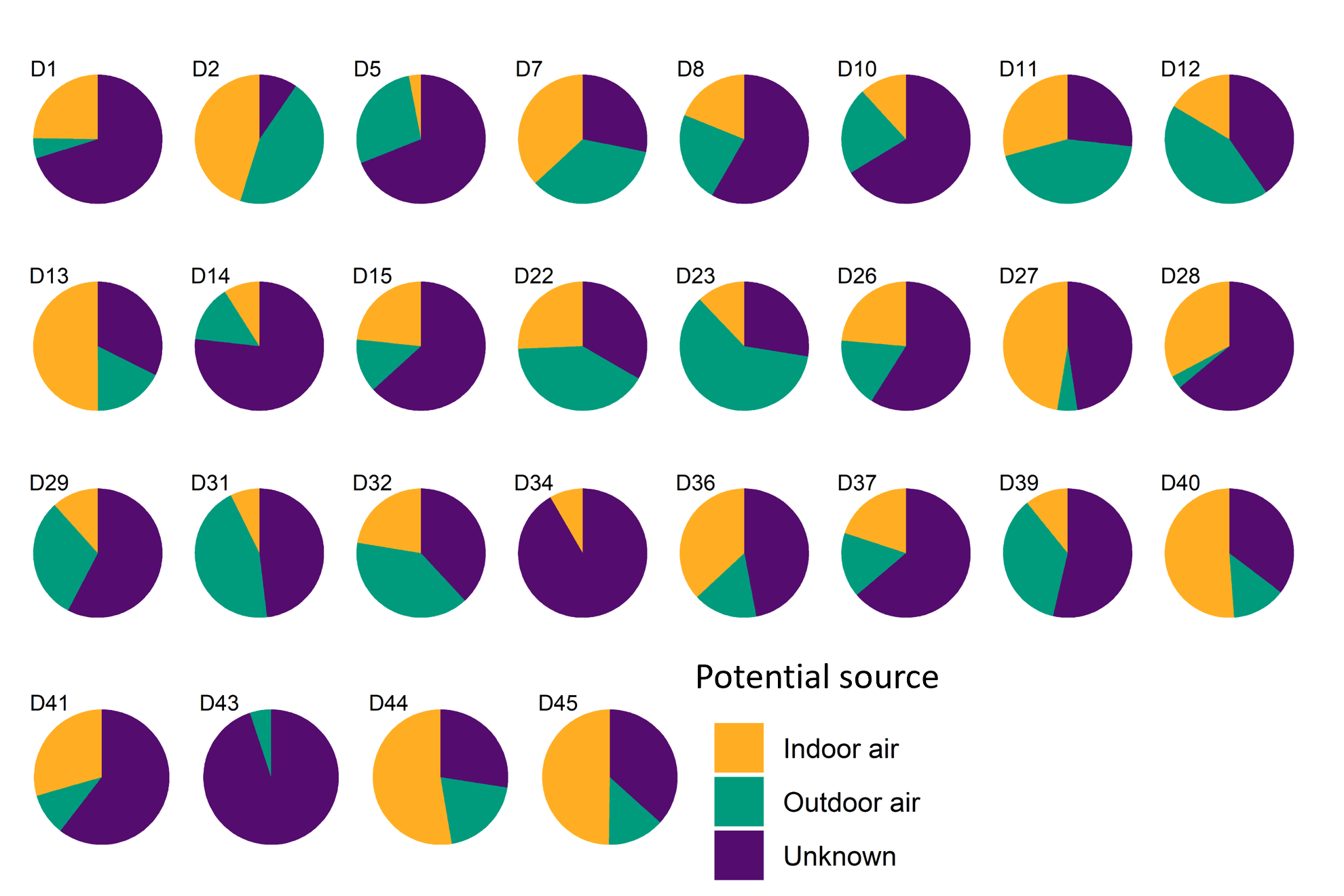

Supplement: Supplemental Information 4 — Sources were labelled as indoor air and outdoor air. Where ASVs didn’t fall in to these categories, they were labeled as coming from an unknown source. [file peerj-11-16355-s004.png]
